# Association of common variation in the *PPARA *gene with incident myocardial infarction in individuals with type 2 diabetes: A Go-DARTS study

**DOI:** 10.1186/1478-1336-3-4

**Published:** 2005-11-25

**Authors:** Alex SF Doney, Bettina Fischer, Simon P Lee, Andrew D Morris, Graham Leese, Colin NA Palmer

**Affiliations:** 1 The Institute of Cardiovascular Research, Ninewells Hospital and Medical School, Dundee, DD1 9SY, Scotland, UK; 2 Biomedical Research Centre, Ninewells Hospital and Medical School, Dundee, DD1 9SY, Scotland, UK; 3 Division of Medicine and Therapeutics, Ninewells Hospital and Medical School, Dundee, DD1 9SY, Scotland, UK

## Abstract

**Background:**

Common variants of the *PPARA *gene have been found to associate with ischaemic heart disease in non diabetic men. The L162V variant was found to be protective while the C2528G variant increased risk. L162V has also been associated with altered lipid measures. We therefore sought to determine the effect of *PPARA *gene variation on susceptibility to myocardial infarction in patients with type 2 diabetes. 1810 subjects with type 2 diabetes from the prospective Go-DARTS study were genotyped for the L162V and C2528G variants in the *PPARA *gene and the association of the variants with incident non-fatal myocardial infarction was examined. Cox's proportional hazards was used to interrogate time to event from recruitment, and linear regression for analysing association of genotype with quantitative clinical traits.

**Results:**

The V162 allele was associated with decreased risk of non-fatal myocardial infarction (HR = 0.31, 95%CI 0.10–0.93 p = 0.037) whereas the C2528 allele was associated with increased risk (HR = 2.77 95%CI 1.34–5.75 p = 0.006). Similarly V162 was associated with a later mean age of diagnosis with type 2 diabetes and C2582 an earlier age of diagnosis. C2528 was also associated with increased total cholesterol and LDL cholesterol, which did not account for the observed increased risk. Haplotype analysis demonstrated that when both rare variants occurred on the same haplotype the effect of each was abrogated.

**Conclusion:**

Genetic variation at the *PPARA *locus is important in determining cardiovascular risk in both male and female patients with diabetes. This genotype associated risk appears to be independent of the effect of these genotypes on lipid profiles and age of diagnosis with diabetes.

## Background

Dysregulation of fatty acid metabolism plays a pivotal role in the aetiology of type 2 diabetes [1], and explains, at least in part, the association between obesity, type 2 diabetes and cardiovascular disease (CVD). PPARα is a member of the nuclear receptor super-family of ligand-activated transcription factors. Ligands for PPARα include polyunsaturated fatty acids and the fibrate class of lipid-lowering drugs [2]. It is expressed at high levels in several cell types involved in the atherosclerotic process [3], and its activation has beneficial effects on plasma lipids, endothelial function and markers of inflammation [4]. Thus, the *PPARA *gene is a strong candidate for a genetic determinant of CVD risk in people with type 2 diabetes [5].

The *PPARA *gene has been screened for common variation [6-8]. The most studied variant is the leucine 162 valine (L162V) polymorphism, present at allele frequencies between 5 and 10%, and situated in the DNA binding domain. Functional studies have demonstrated that the V162 allele is more active *in vitro *[7,9], and the V162 allele has been associated with altered plasma lipid levels [6,8,10], delayed progression of angiographically determined CV disease in the Lopid Coronary Angiography Trial (LOCAT), and reduced risk of ischemic heart disease in the Second Northwick Park Heart Study (NPHS2) [11]. A second, more common, G→C variant situated in intron 7 (G2528C) is in partial allelic association with the L162V variant and shows opposing effects on cardiovascular risk and cardiac growth [9,11]. Recently it has been demonstrated that haplotypes of these variants in association with a further A→C variant in intron 1 influence age of onset of type 2 and time to requiring insulin [12].

PPARα activators improve the dyslipidemia associated with type 2 diabetes and may be particularly beneficial in lowering risk of CVD in subjects with type 2 diabetes or metabolic syndrome [13]. We therefore investigated the association between *PPARA *gene variation with risk of CVD and diabetes related traits in Caucasian subjects with type 2 diabetes participating in the prospective population-based Genetics of Diabetes Audit and Research in Tayside Scotland (Go-DARTS) study [14-16].

## Results

The clinical characteristics of the genotyped cohort are shown in table 1. The allele frequencies of both polymorphisms were consistent with those previously published for European non-diabetic populations (table 2). The two polymorphisms were both in Hardy-Weinberg equilibrium and were in significant linkage disequilibrium D' = 0.204 p =< 0.00001. Estimated haplotype frequencies indicated very similar values with those previously published (table 3). There was little evidence that the genotypes either singly, or when included in the model together, were associated with blood pressure or lipid measurements (table 4). V162 was associated with a generally more cardioprotective profile with V/V homozygotes having lower systolic and diastolic blood pressure, lower LDL cholesterol and higher HDL cholesterol than L/L homozygotes, although none of these differences were significant. Conversely C2528 homozygotes had a small but significantly higher total cholesterol and calculated LDL cholesterol compared to G/G homozygotes. We also found an association of genotype with age diagnosed with type 2 diabetes, with the V162 allele being associated with a significantly later age of diagnosis and the C2528 allele with a significantly earlier age of diagnosis (Table 5). When we considered haplotypes we found that V162-G2528 was associated with almost a 4 year delay in diagnosis with diabetes compared to the common L162-G2582 haplotype (p = 0.004). This association was completely abrogated when C2528 occurred together with V162 as a haplotype.

**Table 1:** Clinical characteristics of the Go-DARTS cohort

No of individuals	1810 (54% male)
Age at recruitment (years)	63.1 (9.6)
Age at diagnosis	54.9 (9.0)
Body Mass Index (kg/m^2^)	30.5 (5.4)
Insulin treatment	839 (44.1%)
Smoking History	958 (50.4%)
Prevalent cerebrovascular disease	67 (3.5%)
Prevalent angina	178 (9.4%)
Previous myocardial infarction	323 (17.0%)

Data shown are mean (SD) for continuous variables and n (%) for categorical variables.

**Table 2:** *PPARA *genotype distribution and allele frequencies in the Go-DARTS cohort. The corresponding allele frequencies from the Second Northwick Park Heart Study (NPHS2)^11 ^is shown for comparison.

		**n**			**n**
**L162V**	L/L	1573	**G2528C**	GG	1216
	L/V	224		GC	529
	V/V	13		CC	64
		1810			1809
**Go-DARTS allele freq.**		0.069 (0.061–0.077)			0.182 (0.169–0.194)
**NPHS allele freq.**		0.063			0.174

**Table 3:** Estimated Haplotype frequencies in Go-DARTS. Frequencies in NPHS2 are given for comparison

**Haplotype**	**Go-DARTS**	**NPHS2**
**L162-G2528**	0.802	0.804
**L162-C2528**	0.130	0.132
**V162-G2528**	0.016	0.021
**V162-C2528**	0.052	0.041

**Table 4:** Biochemical parameters at genotyping. Mean and 95% confidence intervals of all readings taken within 2 years prior to enrolment in study

	**L162V**					
	**L/L**		**L/V**		**V/V**	
**BMI**	30.5	30.2–30.7	30.7	30.0–31.4	28.9	26.0–31.9
**SBP mmHg**	142.5	141.7-141.3	141.3	139.3–143.3	134.9	126.7–143.1
**DBP mmHg**	79.5	79.1–79.8	79.7	78.6–80.7	77.3	73.1–81.5
**Cholrat† mmol/L**	4.5	4.4–4.6	4.8	4.5–5.0	4.3	3.3–5.3
**Chol mmol/L**	5.2	5.2–5.3	5.3	5.1–5.4	5.3	4.8–5.8
**Trigs mmol/L**	2.7	2.6–2.8	2.8	2.6–3.1	2.4	1.3–3.5
**HDL mmol/L**	1.22	1.20–1.24	1.23	1.20–1.28	1.31	1.13–1.50
**LDL mmol/L**	2.89	2.84–2.92	2.89	2.78–3.00	2.92	2.42–3.41
	**G2528C**					
	**G/G**		**G/C**		**C/C**	

**BMI**	30.5	30.2–30.8	30.4	29.9–30.8	31.40	30.1–32.7
**SBP mmHg**	142.5	141.7–143.4	142	140.7–143.3	140.2	135.5–143.9
**DBP mmHg**	79.5	79.0–79.9	79.7	79.0–80.3	78.2	76.3–80.1
**Cholrat† mmol/L**	4.49	4.37–4.60	4.74	4.57–4.91	4.51	4.03–4.98
**Chol mmol/L**	5.20	5.15–5.25	5.26	5.18–5.34	5.56	5.33–5.79*
**Trigs mmol/L**	2.73	2.62–2.83	2.75	2.59–2.91	2.65	2.18–3.12
**HDL mmol/L**	1.22	1.20–1.24	1.23	1.20–1.25	1.25	1.17–1.34
**LDL mmol/L**	2.88	2.83–2.92	2.88	2.81–2.95	3.22	3.01–3.44*

*P < 0.05 ANOVA co-dominant model

† Cholrat = Total cholesterol/HDL Ratio

**Table 5:** Influence of genotype and inferred haplotypes on age diagnosed with type 2 diabetes.

	**Age diagnosed**		
	Beta	95% CI	p

V162*	2.6	0.2–5.1	0.034
**C2528†**	-1.1	-2.0–0.2	0.022
			
**Haplotype**			
**L162-G2582**	Ref		
**L162-C2528**	-0.40	-1.25 – 0.45	0.36
**V162-G2528**	3.89	1.26 – 6.51	0.004
**V162-C2528**	-0.28	-1.65 – 1.10	0.69

*Co-dominant model

† Dominant model

Both variants included in the model

During a median follow up time 49.6 months there were 108 non-fatal myocardial infarction events and 355 deaths from all causes. In a fully adjusted Cox's proportional hazards model (table 6) we found that V162 was significantly protective against non-fatal myocardial infarction (HR 0.31, 95%CI 0.10–0.93, p = 0.037), while the C2528 variant was associated with a significantly higher risk of non-fatal myocardial infarction (HR 2.77, 95%CI 1.34–5.75, p = 0.006). This association was found to be similar in both sexes. Neither variant demonstrated any evidence of an association with risk of myocardial infarction when considered in isolation. Again, when we considered haplotypes, we found that compared to the haplotype with both common alleles, the haplotype L162-C2528 was associated with a significantly increased cardiovascular risk (HR 1.68 95%CI 1.16–2.43 p = 0.006) and the V162-G2528 a decreased risk although in this case this was not significant (HR 0.54, 95%CI 0.20–1.48, p = 0.23). Again the relative associations of each variant were completely abrogated when they occurred together on the same haplotype. The inclusion of total cholesterol in the model did not attenuate these observed associations but rather further strengthened them (V162: HR 0.28, 95% CI 0.09–0.89, p = 0.031 and C2528: HR 2.87, 95%CI 1.38–5.95, p = 0.005) demonstrating that the increased risk associated with the C2528 was not linked to raised cholesterol levels. When a combined endpoint of death from all cause, and non-fatal myocardial infarction was considered in the same model it was found that the V162 continued to demonstrate a reduced risk of an event although the association was attenuated (HR 0.52, 95%CI 0.28–0.98, p = 0.044). C2528 again demonstrated an increased risk although this was now weak and borderline non-significant (HR 1.52, 95% CI 0.99–2.31, p = 0.052).

**Table 6:** Prospective model of *PPARA *variants and non-fatal myocardial infarction risk in the Go-DARTs cohort. A full set of data was available on 1806 individuals, 108 recorded non fatal myocardial infarctions during the period of observation, with a total of 94497.6 months of observation. Both *PPARA *variants were analysed using a co-dominant model.

	**Hazard Ratio**	**95% CI**	**P**
**V162**	0.31	0.10 0.93	0.037
**C2528**	2.77	1.34 5.75	0.006
**Smoking**	1.39	0.93 2.10	0.112
**Gender**	0.72	0.48 1.08	0.107
**Age at recruitment**	1.05	1.02 1.07	<0.001
**Insulin treatment**	2.56	1.69 3.89	<0.001
**Prevalent angina**	5.64	3.80 8.40	<0.001
**Prevalent cerebrovascular disease**	1.29	0.67 2.51	0.445
**Prevalent myocardial infarction**	3.90	2.60 5.81	<0.001
			
**Haplotypes**			
**L162-G2582**	Ref		
**L162-C2528**	1.68	1.16–2.43	0.006
**V162-G2528**	0.54	0.20–1.48	0.23
**V162-C2528**	0.96	0.48–1.94	0.91

## Discussion

It has been previously demonstrated that two common variants at the *PPARA *locus are associated with opposing risks of development of atherosclerotic vascular disease and myocardial infarction in two separate populations of non-diabetic male subjects taking part in the LOCAT and NPHS2 studies [11]. Individuals with type 2 diabetes are however particularly susceptible to atherosclerotic macrovascular disease, and PPARα activators such as the fibrate group of drugs appear to be particularly beneficial in reducing cardiovascular events in this group of patients [13]. In this study we have confirmed the observation that V162 is associated with a decreased risk and the C2528 variant is associated with an increased risk of cardiovascular disease and that this observation can be extended to individuals with type 2 diabetes. We also found that the association is similar in both male and female individuals. Finally we confirm a recent finding that these variants are associated with opposing influences on age of diagnosis with type 2 diabetes [12], and that the C2528 variant is also associated with significantly higher total cholesterol and calculated LDL cholesterol levels.

Several studies have considered the potential clinical importance of genetic variation at the *PPARA *locus although most have concentrated on lipid levels and have considered the L162V variant in isolation. These studies have been inconsistent indicating that L162V may influence levels of cholesterol or other lipoproteins, depending on the population analysed [6,8,10,12,17,18], while other studies have found no evidence for such an association [19,20]. These inconsistencies may be due in large part to differing environments, genetic background and diseased status (including medications prescribed) between the populations considered. For instance the relative concentration in the diet of saturated to polyunsaturated fat has recently been demonstrated to significantly affect association of L162Vgenotype with lipoprotein profile [21,22]. Furthermore, it is likely that there will be differential usage of fibrate (as well as other lipid modifying) drugs between individuals with type 2 diabetes and non-diabetic populations which may also influence the lipid levels differentially by PPARα haplotype [23,24]. Gene/gene interactions may also be important as evidenced by the observation that variants in the *PPARD *and *APOE *genes can influence the observed association [10,20]. The inevitability of gene/environment interactions, and the observed inconsistency between studies, illustrate the difficulties of considering single measures of quantitative traits. Such measures are likely to vary considerably over an individual's lifetime, depending on health status and diet. Importantly, the clinical measures in this study were a mean of multiple measures taken over up to a three year period and therefore represent a limited integration of such temporal fluctuations.

In this study we found that the G2582C variant, but not the L162V variant, influenced lipid levels and this association was not influenced by L162V. The difference between the mean values of LDL cholesterol for GG individuals compared to CC was rather small (0.36 mmol/L) and even in this high risk population did not account for the increased cardiovascular risk associated with C2582. This was not unexpected, as in keeping with the previous studies in non-diabetic men, inclusion of total cholesterol or LDL cholesterol in the model did not affect the association with cardiovascular outcomes, indicating that the increased risk associated with the C2528 variant is not likely to be through its influence on lipid levels.

Few studies have considered cardiovascular disease or considered variants other than the L162V. One recent study also demonstrated a non significant trend towards a protective effect of V162 in individuals with diabetes [19]. This study did not consider the G2825C variant. The present study however confirms that the V162 variant is protective against nonfatal myocardial infarction, while the C2528 variant is associated with an increased risk in this population with type 2 diabetes. These observations also appear to affect overall mortality in this population. This apparent consistency across studies with respect to cardiovascular events probably reflects the small, though global, modulation in phenotype acting across an individual's lifetime due to the slight changes in activity of PPARα associated with each variant. Unlike single measures of lipids or lipoproteins, this is less likely to be effected by temporal environmental changes. This is also likely to be true for clinical events such as age of diagnosis. In the previous study that considered age of diagnosis a further variant in intron 1 of *PPARA *was used to construct haplotypes with L162V and G2825C [12].

In this study, as in ours, the C2825 was associated with an earlier age of diagnosis, with the V162 allele being associated with protection from early diagnosis, in a manner consistent with the modulation of CVD risk. This is the first study to present the association with age-of-diagnosis of type 2 diabetes in the same population with the association with cardiovascular risk, and we can state that inclusion of age of diagnosis does not modulate the observed associations with cardiovascular risk and vice-versa, demonstrating that these are independent observations. This is not surprising, as *PPARA *variation is associated with CVD risk regardless of diabetic status.

The G2825C variant is in a non-coding region (intron 7) and therefore unlikely to be a directly causal variant, however several other studies have indicated its potential biological importance [9,11,12] and also a reduced response to fibrate therapy [25]. These observations together with that of PPARα activation through fibrate therapy have given rise to the suggestion that the C2528 variant is associated with reduced RNA transcription and hence lower PPARα levels. Although this suggestion together with a mechanism remains to be demonstrated, it is probably due to a further (as yet) unidentified variant in or near the *PPARA *gene.

We have previously demonstrated a similar situation in the *PPARG *locus in which the biological effect of a variant with probable functional consequences is consistently modified in an opposing manner by the presence of a variant for which a function is difficult to ascribe [14,15]. Given the role of the PPARs as master controllers of energy metabolism it is likely, as the accumulating evidence appears to suggest, that they will manifest epistasis and balanced polymorphism allowing for rapid adaptive evolutionary response to widely differing environmental challenges. Indeed, the existence of widespread, balanced variation has recently been suggested for genes involved in complex traits [26].

## Conclusion

We have confirmed the potential importance of genetic variation at the *PPARA *locus in modulating susceptibility to cardiovascular disease, and have shown that this association is relevant to individuals with type 2 diabetes.

## Methods

In the Tayside region of Scotland detailed clinical information on all individuals with diabetes mellitus is recorded on a continuously updated electronic clinical information system known as DARTS (Diabetes Audit and Research in Tayside Scotland) [16]. Validated, region-wide electronic record-linkage techniques facilitate the identification of individuals with diabetes in the Tayside population with a sensitivity of 97% [16]. Relevant clinical data is linked to databases containing all inpatient hospital admissions in Tayside from 1980 with diagnostic codes from ICD-9/10 (International Classification of Diseases, ninth and tenth revisions), and records of death certificates from the registrar general. This automated electronic follow-up is manually validated through a continuous cycle of review by dedicated study clinicians. Incident cardiovascular events in this population have been described previously [27].

Following written informed consent from individuals registered on DARTS, blood samples for genetic studies have been collected, thereby creating a genetic sub-study known as Go-DARTS. Rigorous compliance with NHS data protection and encryption standards is maintained and the study was approved by the local research ethics committee.

The PPARα L162V and G2528C genotypes were determined in 1,810 individuals all of whom were Caucasian with type 2 diabetes diagnosed between the age of 35 and 70 years. Taqman (Applied Biosystems) allelic discrimination assays were used. The primers and probes used for the allelic discrimination assays were as follows: L162V Forward primer-CAGAAACAAATGCCAGTATTGTCG, Reverse primer-GGCCACCTTACCTACCGTTGTG, L162 probe (FAM labelled) – TTCACAAGTGCCTTTCTGTCGGGATGT, V162 probe (TET labelled) – TTCACAAGTGCGTTTCTGTCGGGATGT.

G2528C Forward primer-TCCTTAAATATGGTGGAACACTTGAAG, Reverse primer-TCACAACCACCAGTTTTGCAT, G2528 probe (FAM labelled) – ATATCTAGTTT**G**GATTCAAAAGCTTCATTTCCCA, C2528 probe (TET labelled) – ATATCTAGTTT**C**GATTCAAAAGCTTCATTTCCCA.

### Statistics

For each clinical measure the mean was determined from multiple measures obtained up to a maximum period of three years (up to two years prior to enrolment, and up to one year following enrolment). LDL cholesterol was estimated through the use of the Freidwald equation. Linear regression was used to determine the association of genotype with each measure corrected for age at genotyping. For determining the association of genotype with age of diagnosis, this was corrected for gender and presence of a history of smoking by determining residuals and adding these to the overall mean age of diagnosis. Cox's proportional hazards was used to model time to first event. All individuals were followed from the point of genotyping until a non-fatal myocardial infarction occurred, or a composite of non-fatal myocardial infarct or all cause death. Censoring occurred either at the end of the study or death from any cause. Both variants were included in the model and it was found that a co-dominant model for each variant produced the best fit. The following variables were also included in the model; age at genotyping, gender, history of smoking, treatment with insulin, a previous history of a myocardial infarction, prevalent angina and prevalent cerebrovascular disease. Haplotype frequency estimates together with haplotype effects were determined using the THESIAS Program [28,29]. In the case of survival analysis by haplotype using THESIAS only age at genotyping, prevalent angina and a previous history of a myocardial infarction were included in the model.

## List of abbreviations

DARTS Diabetes Audit and Research in Tayside Study

Go-DARTS Genetics of DARTS

PPARα Peroxisome Proliferator Activated Receptor alpha

*PPARA *Gene for PPARα

*PPARD *Gene for PPARδ

CVD Cardiovascular disease

LOCAT Lopoid Coronary Angiography Trial

NPHS2 Second Northwick Park Heart Study

HR Hazard Ratio

CI Confidence Interval

## Competing interests

The author(s) declare that they have no competing interests.

## Authors' contributions

ASFD and CNAP wrote the manuscript and performed the analysis, CNAP and ADM conceived the study and participated in its design and coordination. GL helped draft the manuscript and contributed to the data analysis. BF and SL performed the genotyping. All authors read and approved the final manuscript.
